# DNA Methylation-Mediated Overexpression of *CXCL1* in *Helicobacter pylori*-Induced Gastric Cancer: In Silico- and In Vitro-Based Identification of a Potential Biomarker for Carcinogenesis

**DOI:** 10.3390/ijms24010795

**Published:** 2023-01-02

**Authors:** Jibran Sualeh Muhammad, Shaista Manzoor, Zheng-Guo Cui, Ghalia Khoder

**Affiliations:** 1 Department of Basic Medical Sciences, College of Medicine, University of Sharjah, Sharjah 27272, United Arab Emirates; 2 Department of Environmental Health, University of Fukui School of Medical Sciences, Fukui 910-1193, Japan; 3 Department of Pharmaceutics and Pharmaceutical Technology, College of Pharmacy, University of Sharjah, Sharjah 27272, United Arab Emirates

**Keywords:** *Helicobacter pylori*, epigenetics, DNA methylation, gastric cancer, *CXCL1*, biomarker

## Abstract

Given the high global prevalence and mortality associated with gastric cancer, and its known causal link with *Helicobacter pylori* infection, it is important to have a biomarker to identify malignant transformation at early stages. Previously, we, and others, have reported that *H. pylori*-induced epigenetic changes could mediate carcinogenic transformation of the gastric cells. Also, CXCL1 secreted by gastric cancer cells was reported as a key diagnostic and prognostic biomarker for the pathogenic progression of gastric cancer. In this study, for the first time, we aimed to investigate the role of *H. pylori*-induced DNA methylation-based epigenetic regulation of *CXCL1*. In silico analysis of publicly available datasets and in vitro experiments were performed. Our results showed that *CXCL1* is highly expressed in both gastric cancer tissues and gastric cancer cells infected with *H. pylori*. Further, we showed and confirmed that *H. pylori*-mediated overexpression of *CXCL1* is due to hypomethylation of its promoter region. Since epigenetic events such as DNA methylation happen early in the sequence; *H. pylori*-induced *CXCL1* hypomethylation could likely be detected at an early stage of gastric cancer development. Epigenetic modifications, such as *CXCL1* hypomethylation, are reversible and could potentially be a therapeutic target using demethylation drugs.

## 1. Introduction

According to the International Agency for Research on Cancer (IARC), gastric cancer was the fifth-most diagnosed cancer type and was responsible for 7.7% of all cancer-related deaths in 2020 [1]. One of the most important causes of the development of sporadic gastric cancer is chronic *Helicobacter pylori* infection. Based on this knowledge, the IARC reconfirmed *H. pylori* as a class I carcinogen causing gastric cancer [2]. Most of the time, *H. pylori* infection is acquired during childhood and then it usually persists for life unless treated. The prevalence of *H. pylori* infection exceeds 50% in adults from many well-developed countries; however, the associated gastric, as well as non-gastric diseases only develop in 20% of the infected individuals [3]. Chronic infection with *H. pylori* accounts for approximately 89% as the cause of gastric cancer cases worldwide, but this percentage varies greatly between places around the world [4,5]. Hence, for decades, the specific mechanism by which *H. pylori* infection causes gastric cancer has remained a mystery.

*H. pylori*-induced damage to the gastric mucosal layer is initiated with the infiltration of inflammatory immune cells. This is followed by *H. pylori*-associated acute neutrophilic gastritis, which progresses to chronic gastritis. Even after successful *H. pylori* eradication therapy, a significant number of these inflammatory cells persist in the gastric mucosa and hamper the healing process [6]. The activation and migration of these inflammatory cells in the tumor microenvironment of *H. pylori*-infected gastric mucosa are thought to be related to the increased expression and release of various cytokines from the gastric epithelial cells. These cytokines are now proven to be a definite immunomodulator, attracting and activating more immune cells, leading to long-lasting inflammatory effects [7]. Recent studies have demonstrated that mucosal levels of interleukins and tumor necrosis factor were significantly higher in *H. pylori*-infected patients [8]. Previously, we also studied the secretion of the cytokine interleukin-8 (IL-8) from *H. pylori*-infected gastric epithelial cells and showed that its inhibition could be cytoprotective [9]. However, the roles of the chemokines, which are the chemotactic cytokines, and their receptors in tumorigenesis need further exploration.

High chemokine levels were reported to be a poor prognostic factor in studies using human samples from several types of cancer, including gastric cancer [10]. The C-X-C chemokine family of genes, which is located on chromosome 4 and includes CXC motif chemokine ligand 1 (*CXCL1*), is one of the most studied CXC chemokines that activate the CXC motif chemokine receptor 2 (CXCR2). CXCL1 and CXCR2 are known to have a regulatory role in tumor cell migration, invasion, and metastasis in a variety of cancers [11]. In gastric cancer as well, *CXCL1*-overexpressing tumor cells showed increased migratory and invasive potential, whereas *CXCL1* depletion significantly decreased the carcinogenic activities [12]. Suggesting that an invasive mechanism exists by which CXCL1-CXCR2 could contribute to the progression of gastric cancer.

It has been reported that the accumulation of epigenetic abnormalities in *H. pylori*-infected gastric mucosa promotes the development of gastric cancer [13,14]. Nowadays, a high number of epigenetic biomarkers are being proposed; hence, it is the right time to identify and adopt epigenetic marks as a reliable biomarker for cancer diagnosis [15]. In this study, for the first time, we aimed to investigate whether *H. pylori* infection induces DNA methylation-based epigenetic modulation of *CXCL1*, which could be utilized as a potential epigenetic biomarker in the early detection of *H. pylori*-induced gastric carcinogenesis.

## 2. Results

### 2.1. Differentially Expressed Genes (DEGs) Detected from Human Gastric Cancer Tissue Samples and H. pylori-Infected Cells Compared to Normal

**Figure 1:**
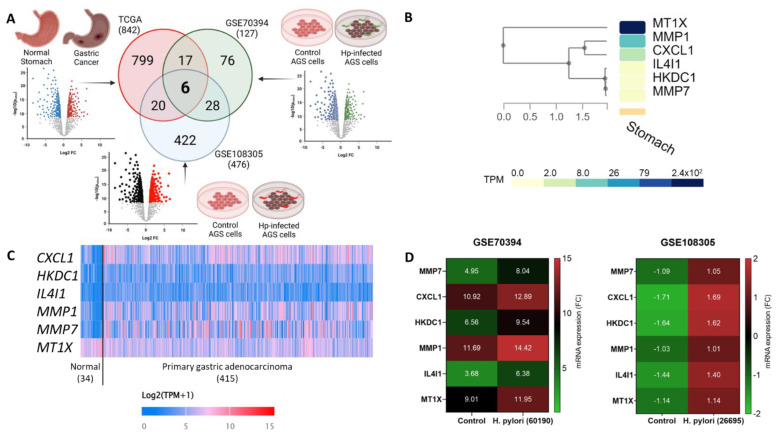
Differentially expressed genes (DEGs) detected from gastric cancer and *H. pylori*-infected cells compared to healthy tissues or control cells. (**A**) Filtering out DEGs by comparing three datasets, the cancer genome atlas (TCGA) for human gastric tissues and two datasets of *H. pylori*-infected AGS cells from Gene Expression Omnibus (GEO); (**B**) Heatmap derived from GTEx showing expression of six candidate genes in the normal human stomach; (**C**) Heatmap showing the expression patterns of the six candidate genes in normal versus primary gastric adenocarcinoma (TPM: Transcripts per million); (**D**) Relative mRNA expression of the six candidate genes in *H. pylori*-infected compared versus the expression data of the same gene from uninfected gastric epithelial cells. The black bars represent mRNA expressions from *H. pylori*-infected AGS cells compared to the untreated cells from GSE108305, and the white bars represent *H. pylori*-infected AGS cells compared to the untreated cells from GSE70394. All the gene expressions reported are statistically significant in comparison to the healthy controls or uninfected cells (*p* < 0.001).

To identify the key DEGs in *H. pylori*-induced gastric cancer tissues, we used three different datasets. From these datasets, a volcano plot was generated to get the up- and down-regulated genes, and a Venn diagram was drawn to identify the common genes. Comparing normal stomach tissue with primary gastric adenocarcinoma from the Cancer Genome Atlas (TCGA) database produced 842 DEGs. AGS control cells versus those infected with *H. pylori* strain 26695 (GSE108305) showed 476 DEGs. Whereas, *H. pylori* strain 60190 infected AGS cells compared to control cells (GSE70394) showed 127 DEGs. Comparative analysis of these three datasets identified six candidate genes, *CXCL1*, *HKDC1*, *IL4I1*, *MMP1*, *MMP7*, and *MT1X* (Figure 1A). Analysis of gene expression in a normal human stomach from the GTEx database showed that *MT1X* and *MMP1* are highly expressed in normal stomach tissue (>8.0 transcripts per million (TPM)), *CXCL1* has a medium expression (~2.0 TPM), and *HKDC1*, *IL4I1*, and *MMP7* are barely expressed (~0 TPM) (Figure 1B). Detailed analysis of the TCGA database showed significant upregulation of the five genes, except for *MT1X*, which was downregulated. However, all six genes were upregulated in AGS cells infected with both *H. pylori* strains compared to gene expressions in control AGS cells (Figure 1D). *CXCL1* and *MMP7* showed the most significant and highest differential upregulation in gastric cancer samples compared to the healthy stomach (Supplementary Figure S1). *MT1X* is also highly expressed in normal stomach tissues, but *CXCL1*, having a stronger association with gastric carcinogenesis, and showing a lower expression in normal tissue, came out as a potential molecule for further analysis.

### 2.2. CXCL1 Interacting Proteins and Pathways Analysis

**Figure 2:**
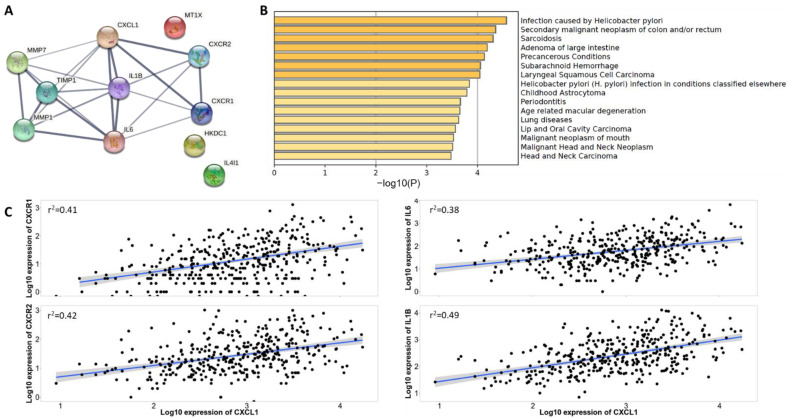
CXCL1 protein–protein interactions and functional pathway enrichment analysis. (**A**) Interactions of CXCL1 with protein molecules transcribed by the other 5 candidate genes; (**B**) Functional pathways enrichment output obtained when protein molecules transcribed by the six candidate genes and other closely interacting proteins were used as input; (**C**) Co-expression analysis of *CXCL1* with *CXCR1* (Spearman correlation coefficient r^2^ = 0.41), *CXCR2* (r^2^ = 0.42), *IL6* (r^2^ = 0.38) and *IL1B* (r^2^ = 0.49) in human gastric cancer tissue samples (*n* = 375). All the values are statistically significant (*p* < 0.001).

Next, we sought to perform a protein–protein interaction analysis of the proteins translated by the six candidate genes. Three of the candidate molecules (CXCL1, MMP1, and MMP7) showed a close association and the other three (HKDC1, IL4I1, and MT1X) showed no interactions. Moreover, upon adding the first shell of interaction along with the query proteins, we found a very strong interaction (edge confidence > 0.7) of CXCL1 with CXCR1, CXCR2, IL6, and IL1B (Figure 2A). Interestingly, functional pathway enrichment of the three closely interacting candidate proteins and four strongly interacting first shells of proteins confirmed their association with *H. pylori* infection, malignant transformation, and precancerous conditions, among others (Figure 2B). Next, the genetic co-expression analysis showed a significant and positive correlation between CXCL1 overexpression and upregulation of CXCR1 (r^2^ = 0.41), CXCR2 (r^2^ = 0.42), IL6 (r^2^ = 0.38), and IL1B (r^2^ = 0.49) (*p* < 0.001 for all the co-expressions) (Figure 2C). These results suggested that *H. pylori*-infection-induced increased gene and protein expression of CXCL1 is correlated with the increase in other potent pro-inflammatory cytokines. These molecules together transform the cellular microenvironment towards the precancerous condition and ultimately lead to malignant transformation.

### 2.3. Detailed Analysis of CXCL1 Expression in TCGA Stomach Adenocarcinoma

**Figure 3:**
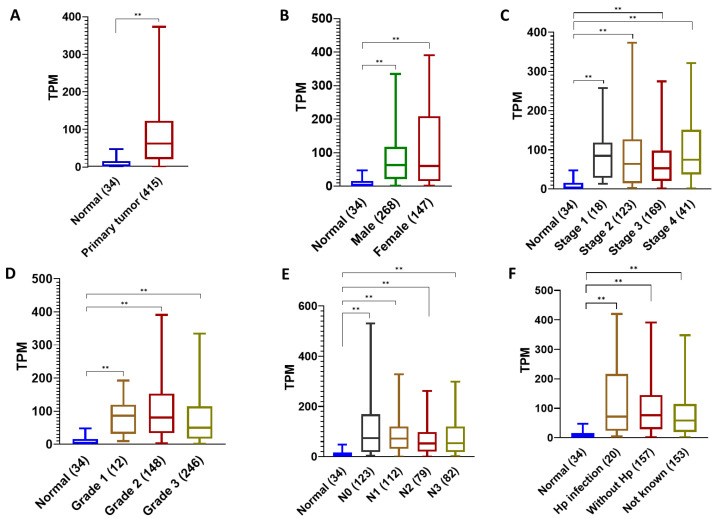
Detailed analysis of *CXCL1* expression in TCGA stomach adenocarcinoma. (**A**) Expression in normal primary tumor samples; (**B**) Gender-wise differences in expression; (**C**) Expression in normal compared to individual cancer stages; (**D**) Expression based on normal versus tumor grade; (**E**) Expression based on metastasis in the lymph nodes; (**F**) Expression in gastric cancer tissue with or without *H. pylori* infection. All the gene expressions reported are statistically significant (** *p* < 0.001). (TPM: Transcripts per million; Hp: *H. pylori*).

To understand the expression pattern of *CXCL1* in gastric carcinogenesis, we performed a detailed analysis on TCGA stomach adenocarcinoma (STAD) samples. Based on sample types, median *CXCL1* expression was 62.59 TPM in the primary tumor and 4.197 TPM in the normal stomach (Figure 3A). Based on the patient’s gender, median *CXCL1* expression was 62.936 TPM in males and 60.708 TPM in females with the primary tumor, and 4.197 TPM in the normal stomach (Figure 3B). Based on individual cancer stages, the median expression of *CXCL1* in stage 1 was 84.585 TPM, in stage 2 it was 63.821 TPM, in stage 3 it was 52.552 TPM, and in stage 4 it was 74.474 TPM (Figure 3C). Based on tumor grade, in grade 1 the median expression was 86.112 TPM, in grade 2 it was 80.484 TPM and in grade 3 it was 49.751 TPM. Based on nodal metastasis, N0 median expression was 73.824 TPM, N1 median expression was 72.788 TPM, N2 median expression was 52.775 TPM, and N3 median expression was 53.076 TPM (Figure 3D). Based on *H. pylori* infection status, the median *CXCL1* expression in *H. pylori*-infected individuals was 72.113 TPM, whereas the median expression in STAD samples without *H. pylori* infection was 76.124 TPM. Patients samples with unknown *H. pylori* infection status showed a median *CXCL1* expression of 58.808 TPM (Figure 3E). These data suggested that *CXLC1* overexpression is directly related to the severity, grade, and stages of STAD. Also, *H. pylori* infection is associated with high *CXCL1* expression compared to expression in normal tissues. The fact that *CXCL1* median expression was higher in grade 1 and stage 1 of STAD is suggestive that *CXCL1* might have a potential role in promoting the early carcinogenic transformation.

### 2.4. Detailed Analysis of CXCL1 Promoter Methylation in TCGA Stomach Adenocarcinoma

**Figure 4:**
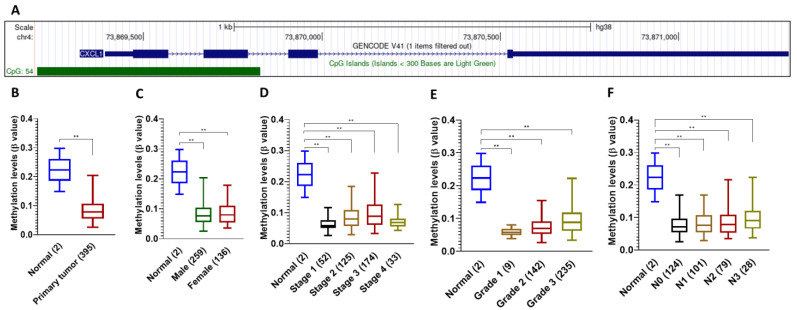
Detailed analysis of *CXCL1* promoter methylation in TCGA STAD samples. (**A**) Location of CpG island in the promoter region of the *CXCL1* gene. (**B**) *CXCL1* promoter methylation based on sample types; (**C**) *CXCL1* promoter methylation based on patient’s gender; (**D**) *CXCL1* promoter methylation based on individual cancer stages; (**E**) *CXCL1* promoter methylation based on tumor grade; (**F**) *CXCL1* promoter methylation based on nodal metastasis. All the gene expressions reported are statistically significant (** *p* < 0.001).

To understand the regulation of *CXCL1* gene expression, we investigated the possibility of DNA methylation-based epigenetic modulation. Searching the genomic sequence of the promoter region of *CXCL1*, we found the presence of a CpG island (Figure 4A). Next, we performed a detailed analysis of *CXCL1* promoter methylation levels in TCGA stomach adenocarcinoma (STAD) samples. Based on sample types, the median *CXCL1* promoter methylation beta value was 0.078 in the primary tumor and 0.223 in the normal stomach (Figure 4B). Based on the patient’s gender, the median *CXCL1* methylation was 0.077 in males and 0.08 in females with primary tumors (Figure 4C). Based on individual cancer stages, the median methylation level of *CXCL1* in stage 1 was 0.058, in stage 2 was 0.079, in stage 3 was 0.089, and in stage 4 was 0.068 (Figure 4D). Based on tumor grade, in grade 1 the median methylation was 0.057, in grade 2 it was 0.069 and in grade 3 it was 0.088. Based on nodal metastasis, N0 median methylation was 0.071, N1 median methylation was 0.076, N2 median methylation was 0.078, and N3 median methylation was 0.091 (Figure 3E). Significant hypomethylation of the *CXCL1* promoter region in primary tumor samples compared to normal, which is consistent with overexpression, suggests DNA methylation-based epigenetic regulation in these samples.

### 2.5. In Vitro CXCL1 Expression, Promoter Methylation, and Protein Secretion

**Figure 5:**
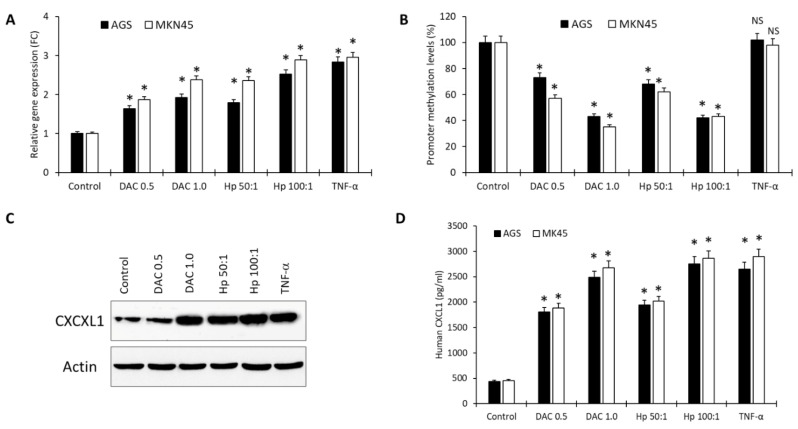
In vitro *CXCL1* gene expression, promoter methylation, protein expression, and protein secretion in *H. pylori*-infected, decitabine (DAC)-treated, or TNFα-treated gastric cells. AGS and MKN45 cells were left untreated (Control) or treated with DAC (0.5 and 1.0 μM), *H. pylori* (MOI 50:1 and 100:1), and TNFα (10 ng/mL). (**A**) *CXCL1* relative gene expression (fold change) (**B**) Percentage of *CXCL1* promoter methylation levels; (**C**) CXCL1 protein expression; (**D**) Human CXCL1 protein secretion in the cell culture supernatant. Three independent experiments were performed and a *t*-test was used to compare treated samples versus control samples (* *p* < 0.01; NS: not significant).

To understand the role of *H. pylori* infection in modulating the promoter DNA methylation, hyper or hypomethylation, we performed several in vitro experiments. AGS and MKN45 cells were treated with decitabine (DAC: a demethylation agent) for 24 h, *H. pylori* (ATCC strain 26695) for 24 h or cells were stimulated with TNFα (positive control) for 30 min. Our results showed that *CXCL1* gene expression significantly increased with all three types of treatment (Figure 5A). Moreover, we found a dose-dependent increase in gene expression when treated with increasing concentration of DAC or increasing multiplicity of infection (MOI) of *H. pylori*. Consistently, we demonstrated that DAC as well as *H. pylori* treatment induces significant hypomethylation of the promoter region of the *CXCL1* gene. However, TNFα treatment did not cause any change in promoter methylation levels compared to untreated cells (Figure 5B). Moreover, our results confirmed the increased CXCL1 protein expression and protein secretion upon treatment with *H. pylori*, DAC as well as TNFα (Figure 5C,D). These data suggested that *H. pylori* infection induces hypomethylation and upregulation of *CXCL1* similar to the effect of a demethylation agent, which induces hypomethylation and overexpression.

### 2.6. CXCL1-Mediated Tumor Microenvironment and Overall Survival in Gastric Cancer Patients

**Figure 6:**
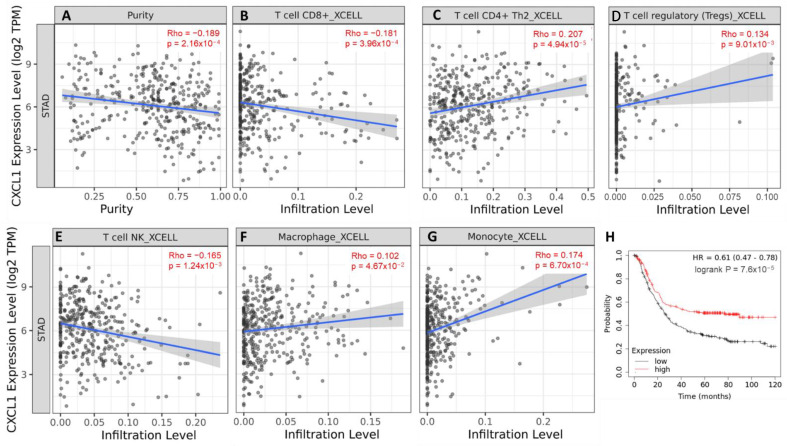
Tumor microenvironment in correlation with *CXCL1* expression and overall patient survival in gastric cancer patients. (**A**) Purity of infiltrating cells; (**B**) T cell CD8+ infiltration; (**C**) T cell CD4+ infiltration; (**D**) Regulatory T cells infiltration; (**E**) NK cell infiltration; (**F**) Macrophages infiltration; (**G**) Monocytes infiltration; (**H**) Kaplan–Meier plot for overall patient probability at risk for over 120 months. (*p* < 0.001).

To study the role of CXCL1 in tumorigenesis, we investigated the infiltrating immune cells in the gastric cancer tumor microenvironment (TME). TCGA STAD samples with significantly good purity were utilized as input cells (Figure 6A). We found that increased expression of *CXCL1* was negatively correlated with CD8+ T cells and NK cells, whereas *CXCL1* expression was positively correlated with CD4+ T cells, T reg cells, macrophages, and dendritic cells (Figure 6B–G). Increased levels of tumor-infiltrating monocytes and macrophages in TCGA STAD samples highly expressing *CXCL1* are suggestive of enhancing tumor invasion and migration. Finally, we created a Kaplan–Meier plot for the same STAD samples and discovered that high *CXCL1* expression is associated with a higher risk probability for a greater number of patients developing serious disease and death (Figure 6H).

## 3. Discussion

Gastric cancer is a highly heterogeneous disease, and long-term *H. pylori* infection is one of the most common causes in most of the world’s population. However, in the majority of cases of *H. pylori* infection, even in patients with similar clinical and pathologic features, carcinogenic transformation happens only in a few [16]. Hence, the identification of a molecule that promotes *H. pylori*-infected gastritis to adenoma and adenocarcinoma would be ideal to be utilized as a biomarker for early diagnosis. For decades, the most accurate means for diagnosing a gastric cancer patient required endoscopic tissue biopsy and then pathological assessment of specific histological features of the tumor [17]. However, the heterogeneity in survival outcomes of patients with the same stage of gastric cancer highlights the need to identify a more accurate molecule for severity risk assessment. In this study, to screen for gastric cancer early diagnostic biomarkers, we utilized a novel in silico strategy using not only the normal versus cancer patient samples but also the *H. pylori*-infected in vitro samples. We have shown that *CXCL1* expression is higher and promoter methylation levels are lower in gastric cancer patients and *H. pylori*-infected gastric cells compared to healthy subjects and control cells.

Cancer arises due to several genetic and epigenetic alterations [18], and many of these happen at very early stages of cancer development. Previously, we and others have reported several DNA methylation-based epigenetic changes due to *H. pylori* infection which could be potential therapeutic targets [13,14,19], but the diagnostic molecules were never identified. In recent years, the usefulness of aberrant epigenetic alterations as biomarkers in cancer cells has been reported [20]. For example, methylation analysis of the Spastic Paraplegia-20 (*SPG20*) gene in various solid tumors as well as paired gastric cancer was reported to be a disease pathogenicity biomarker. These studies highlighted that the *SPG20* promoter region is hypermethylated, and the tumor tissues display a higher methylation heterogeneity compared to the solid tissue normal [21,22]. Based on its stability, frequency, and reversibility, the specific epigenetic alterations could be utilized as a potential candidate for clinically useful cancer biomarkers. For the first time, in this study, we identified that *H. pylori*-induced hypomethylation of the *CXCL1* promoter region is causing its overexpression in infected gastric cells. We showed that increasing *H. pylori* MOI increases the expression and similarly reduces the methylation levels. Furthermore, the DNA methylation-based epigenetic regulation of *CXCL1* was confirmed by a demethylation agent, and interestingly, a similar effect was not seen in TNFα-treated samples.

Gastric cancer tissues show a differential expression of CXCL1 and its receptor CXCR2 [10]. *CXCL1* gene expression was detected in gastric cancer cell lines and gastric cancer mouse tissue [12]. In addition, the expression of *CXCL1* mRNA and protein was higher in human gastric cancer tissue compared with paired non-cancerous gastric tissues [23]. Furthermore, the same studies revealed an upregulation of circulating CXCL1 levels in patients with gastric cancer compared to the healthy controls [10,23]. Also, the expression of CXCR2 was increased significantly in tumor tissue compared with non-cancerous adjacent tissue, which was associated with tumor progression, and more advanced gastric cancer stages [10,24], and the elevated *CXCL1* gene expression was an independent prognostic factor for patient survival [10]. Consistently, our results showed a higher *CXCL1* expression in gastric cancer patient samples, which was positively correlated with *CXCR2* expression.

Although CXCL1 is virtually undetectable at baseline, its expression increases under pathological conditions, and abnormal expression of CXCL1 is often associated with the development of certain cancers [25,26]. We also showed that *CXCL1* expression was minimal in the GTEx database. Moreover, manipulation of CXCL1 expression using monoclonal antibodies has been shown to influence the rates of proliferation, migration, and invasion of human bladder and prostate cancer cells [27]. This is probably because CXCL1 interacts with several immune cells and acts as a chemoattractant, vital for immune response regulation. In a previous study, it was reported that tumor-infiltrating monocytes/macrophages regulate the expression of certain calcium or zinc-binding proteins, which enhances migration and invasion of the cancer cells causing metastases [28]. Here in this study, the gastric cancer microenvironment analysis revealed increased tumor tissue infiltration of monocytes and macrophages which strongly and positively correlated with *CXCL1* expression.

In regards to CXCL1 in other cancers, the level of *CXCL1* expression in colorectal cancer tissues was significantly higher than in adjacent normal tissues and positively correlated with the size of the tumor and its stage of progression [29]. A positive correlation was found between CXCL1 expression compared to the tumor staging, and lymph node metastasis in patients with non-small-cell lung cancer and was associated with poorer disease-free survival and overall survival [29]. It was shown that CXCL1 promoted the survival of breast cancer cells by recruiting myeloid cells in tumors which produce paracrine cytokines to promote tumor survival and metastasis [30]. A higher level of CXCL1 protein expression was found in human bladder cancers with aggressive phenotypes [25]. In osteosarcoma, CXCL1 is known to upregulate the expression of NF-κB via the CXCR2/FAK/PI3K/Akt pathway. This stimulates the increased expression of adhesion molecules promoting aggressiveness of the osteosarcoma and its metastasis abilities [31].

CXCL1 protein levels increased in high-grade malignancies, as well as higher-grade prostate cancer tumors [32]. It has also been suggested that CXCL1 promotes tumor growth in hepatocellular carcinoma (HCC). A high level of *CXCL1* was found in the HCC cancer tissue compared to normal cells. Overexpression of *CXCL1* in HCC cell lines stimulated cell growth, proliferation, and invasion [26]. A recent study indicates that the re–lease of CXCL1 directly contributes to the development of metastases in colorectal cancer [33]. The tumor-associated macrophages-derived CXCL1 is involved in the regulation of breast cancer metastasis by activating the NF-κB/SOX4 pathway [34]. In this study, we indicated that CXCL1 plays a crucial role in the gastric cancer microenvironment, as it might play an essential role in the progress of cancers, mainly by promoting invasion and metastasis, which are the most common causes of cancer progression.

In conclusion, for the first time, in this study, we reported *H. pylori*-induced DNA hypomethylation-mediated epigenetic changes in the *CXCL1* promoter region, which causes an increase in the expression and secretion of CXCL1 from the gastric epithelial cells. In silico analysis showed that *CXCL1* is highly expressed in gastric cancer tissues and gastric cancer cells infected with *H. pylori*. In vitro analysis showed that *H. pylori*-mediated overexpression of *CXCL1* is due to DNA hypomethylation of its promoter region. Protein–protein interaction and co-expression analysis showed a strong association of CXCL1 with CXCR1, CXCR2, IL6, and IL1B in human gastric cancer tissue samples. Hence, there was increased recruitment of monocyte/macrophages in the tumor microenvironment, which probably leads to carcinogenic transformation. Since epigenetic events such as DNA methylation happen early in the sequence; *H. pylori*-induced *CXCL1* hypomethylation can likely be utilized as an early detection biomarker. Moreover, epigenetic modifications, such as *CXCL1* hypomethylation, are reversible and could potentially be a therapeutic target using demethylation drugs.

## 4. Materials and Methods

### 4.1. GEO Data Analysis

We searched for transcriptomic datasets in the Gene Expression Omnibus (GEO) from the National Center for Biotechnology Information (https://www.ncbi.nlm.nih.gov/geo/ (accessed on 25 August 2022)), a publicly available genomics and transcriptomic database. The search terms of *H. pylori* and gastric cells were used to identify datasets for *H. pylori*-infected human gastric cancer cells. Two datasets, GSE108305 and GSE70394, were selected. Both datasets included transcriptomic profiling of the human gastric carcinoma-derived cell line (AGS) infected with *H. pylori*. In the dataset GSE70394, AGS cells were infected with *H. pylori* strain 60190 (ATCC 49503) for 24 h and RNA was extracted from three independent experiments in the dataset GSE108305, AGS cells were infected with *H. pylori* strain 26695 (ATCC 700392) for 4 h and RNA was extracted from two independent experiments. For expression and promoter methylation analysis of the candidate gene in gastric cancer tissues versus normal stomach tissues, the cancer genome atlas (TCGA) online portal was accessed. Data are presented as a Box-Whisker plot, showing gene expression and promoter methylation levels in normal versus tumor samples.

### 4.2. In Silico Analysis for Pathway Enrichment, Protein–Protein Interactions, and Correlation

To identify the underlying functional pathways, gene set enrichment analysis was performed using several ontologies resources [35]. Functional protein–protein interactions were depicted using the GeneMANIA database (http://genemania.org/ (accessed on 26 August 2022)). Using six candidate genes as an inquiry, the protein–protein interaction was analyzed based on different networks including co-expression. The NCBI genome browser was searched for the presence of CpG island in the promoter region of the *CXCL1* gene. Lastly, the relationship between *CXCL1* mRNA expression and the risk of disease development in gastric cancer patients was conducted using the Kaplan–Meier plotter (https://kmplot.com/analysis/ (accessed on 26 Aug 2022)).

### 4.3. Cell Lines and Cell Culture

The AGS and MKN45 gastric cancer cell lines (ATCC, Manassas, VA, USA) were used in this study. Both the cell lines were mycoplasma-free and were authenticated using short tandem repeat (STR) genotyping and were verified to be identical with the STR profile in comparing databases (CLS Cell Lines Service GmbH, Eppelheim, Germany). Cells were maintained in appropriate cell culture media (DMEM or RPMI 1460) supplemented with fetal bovine serum (FBS) (10%) and antibiotics (1%) (Sigma Aldrich, St. Louis, MO, USA) at 37 °C and 5% CO_2_.

### 4.4. H. pylori Culture and Treatment

*H. pylori* (ATCC strain) were cultured in Brucella broth medium (BB) supplemented with FBS (10%) under microaerophilic conditions (5% O_2_, 10% CO_2_, and 85% N_2_ at 37 °C) in a Sanyo-Multigas Incubator, (SANYO Electric Co., Ltd. Tokyo, Japan) with 100% humidity on a gyratory shaker set at 160 rpm. The formula absorbance of 0.1 = 10^8^ bacteria/mL was used to estimate the concentration of bacteria in each culture. *H. pylori* were cultured overnight in BB-FBS-10% under the conditions mentioned above and then washed twice with PBS. The medium RPMI 1640, without antibiotics and FBS, was added finally 1 h before the addition of *H. pylori*. Bacteria were then directly added to cells for the indicated times. AGS and MKN45 cells were treated with 10 ng/mL TNF-α as a positive control for inflammatory condition.

### 4.5. Demethylation Treatment

DNA demethylation treatment was performed using 0.5 and 1.0 μM DAC as described previously [36].

### 4.6. RT-PCR and Methylation-Specific PCR (MSP)

An Allprep DNA/RNA mini kit (Qiagen, Hilden, Germany) was used to extract total RNA and genomic (g) DNA. From 1 μg of total RNA, complementary (c)DNA was synthesized using the QuantiTect Reverse Transcription Kit (Qiagen, Hilden, Germany) according to the manufacturer’s protocol. RT-PCR was performed at specific conditions using 1 μL of cDNA, specific primers (Table 1A), a Promega GoTaq^®^ qPCR Master mix (Promega, Madison, WI, USA), and a Qiagen Rotor-gene qPCR machine (Qiagen, Hilden, Germany). Expression levels of the target gene (*CXCL1*) were normalized to GAPDH expression as an internal control.

For bisulfite modification of the DNA, an aliquot of gDNA (2 μg) was treated with an EpiTect Bisulfite Kit (Qiagen, Hilden, Germany). MSP was conducted using 1 μL of the bisulfite-treated DNA, primers specifically designed for methylated and unmethylated DNA sequence of the promoter region of CXCL1 genes (Table 1B), a Promega GoTaq^®^ qPCR Master mix (Promega, Madison, WI, USA) and a Qiagen Rotor-gene qPCR machine (Qiagen, Hilden, Germany). Fully methylated and fully unmethylated control DNAs were purchased from Qiagen and were used as positive and negative controls, respectively. DNA methylation levels were calculated as described previously [36].

### 4.7. Enzyme-Linked Immunosorbent Assay (ELISA)

AGS cells were treated with *H. pylori*, DAC, or TNFα and 24 h post-treatment supernatant was collected and stored at −80 °C. CXCL1 secretion in the supernatant from the treated cells was analyzed by using the CXCL1 ELISA kit (R&D System, Minneapolis, MN, USA), according to the manufacturer’s instructions. A standard curve using recombinant CXCL1 (R&D System, Minneapolis, MN, USA) was employed to determine CXCL1 concentrations in the control and treated samples and the values were presented in pg/mL.

### 4.8. Western Blotting

AGS or MK45 cells treated were lysed with ice-cold radio-immunoprecipitation assay buffer containing a protease cocktail inhibitor (Sigma Aldrich, St. Louis, MO, USA). Whole-cell lysate protein concentration was quantified using the standard Bradford method (BioRad, Hercules, CA, USA). Lysate aliquots containing 30 µg protein were separated by SDS-PAGE gel electrophoresis and were transferred onto a Polyvinylidene difluoride membrane (BioRad, Hercules, CA, USA). The membrane was blocked with 5% skimmed milk (Sigma Aldrich, St. Louis, MO, USA) for 1 h at room temperature, then washed with T-TBST, and reacted with primary antibody (anti-CXCL1 from Abcam, Cambridge, UK) at 1:1000 dilution overnight at 4 °C. The specific Horseradish Peroxidase-labelled secondary antibodies (Abcam, Cambridge, UK) were then reacted with the membrane at 1:4000 dilutions for 1 h at room temperature. Chemiluminescence was detected using Enhanced Chemiluminescence Western blotting detection reagent (BioRad, Hercules, CA, USA). The primary antibody for actin was used as an internal and loading control.

### 4.9. Statistical Analysis

The significance of differences between the two samples (control vs. treated) was evaluated by using the Student’s *t*-test, and all statistical calculations were performed in GraphPad Prism software version 8, and a *p*-value of <0.01 was considered significant. For in vitro experiments, at least two independent technical and biological replicates were assessed, and pooled data were presented as mean ± SEM. For protein expression blots, one representative image out of at least two independent experiments is presented.

## Figures and Tables

**Table 1:** List and sequences of RT-PCR and MSP primers.

(A)						
Gene	F/R		Sequence (5’ > 3’)		Tm (°C)	Product (bp)
*CXCL1*	F R		GCGCCCAAACCGAAGTCATA ATGGGGGATGCAGGATTGAG		65.2 64.8	70
*GAPDH*	F R		CCAGAACATCATCCCTGCCT CCTGCTTCACCACCTTCTTG		62.4 60.8	185
**(B)**						
**Gene**	**F/R**	**M/U**		**Sequence (5’ > 3’)**	**Tm (°C)**	**Product (bp)**
*CXCL1*	F R	M		TTAATTATGTATAAAAGGGGTTCGC TAAAACTCAACAAACGAATCTAACG	58.9 58.9	119
*CXCL1*	F R	U		TAATTATGTATAAAAGGGGTTTGTGG AAACTCAACAAACAAATCTAACAAC	51.0 54.1	116

(F = forward; R = reverse; M = methylated; U = unmethylated; Tm = Melting temperature).

## Data Availability

Not applicable.

## Supplementary Materials

The following supporting information can be downloaded at: https://www.mdpi.com/article/10.3390/ijms24010795/s1.
