# Supplementary material for: DNA Methylation-Mediated Overexpression of CXCL1 in Helicobacter pylori-Induced Gastric Cancer: In Silico- and In Vitro-Based Identification of a Potential Biomarker for Carcinogenesis
Source: Int J Mol Sci. 2023 Jan 2;24(1):795. doi: 10.3390/ijms24010795 (PMC9820856; doi:10.3390/ijms24010795)
Supplement: Supplementary file 1 [file ijms-24-00795-s001.zip › ijms-2023187-supplementary.pdf]

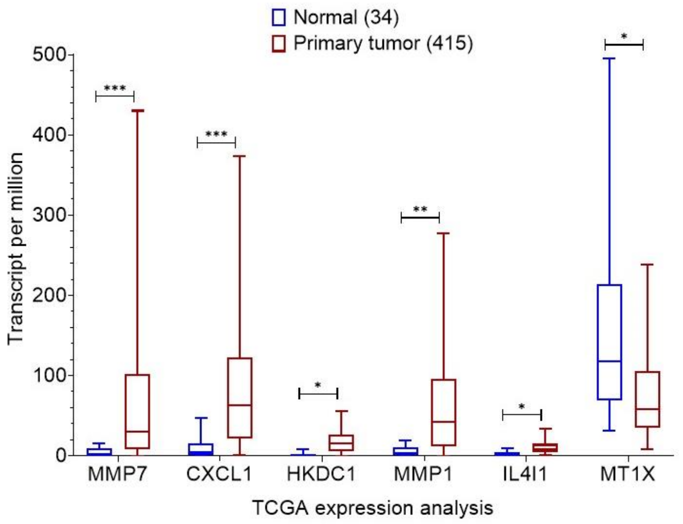

Figure S1. Box plot showing the differential expression of the six candidate genes in normal versus primary gastric adenocarcinoma (TCGA: The cancer genome atlas). All the gene expressions were reported as median, upper quartile, lower quartile, maximum and minimum expression. All comparisons are statistically significant in comparison to the healthy controls (**p* < 0.01; ***p* < 0.001; ****p* < 0.0001).
